# A Word for Our Seamen

**Published:** 1889-02-23

**Authors:** 


### A Word for Our Seamen.

Does Britannia really care for the men by whose aid she rules the waves? Theoretically, in song and sentiment, yes; practically, it was long doubtful. We have been a maritime nation for at least three centuries; yet it is only since this century began that there has been any real attempt to provide for the comfort and welfare, physical and spiritual, of Jack ashore. As if it were not enough that he should face all manner of dangers on the sea, he was allowed, when he came to port and went ashore "with his pockets full of dollars on the spree," to drift into low lodging-houses, where harpies of every sort took advantage of his ready good nature till nearly every penny of his gains had gone to them. Things are better now. In every port of any magnitude there is a Sailors' Home, where he can put up till it is time for him to sail again. These vary in size with the importance of the town in which they are situated; but naturally one of the best belongs to the Port of London.

This Home is the outcome, among other institutions of a more distinctly-charitable nature, of a mission to the seamen of the Port of London which was established in 1825. It was at first called the London Episcopal Floating Church Society, and religious services were held in the ship *Brazen*, which was given by the Admiralty for the purpose. The *Brazen* formed the mission church for twenty years, by which time it was so decayed as to be useless, while the shipping traffic of the Thames had so increased that there was no room for a ship to be permanently moored. The habits of seafaring life also had changed in some degree. Crews were no longer kept on board their ships while they were in port; the men lived on shore, either in lodging-houses or with their families, and a floating church with a chaplain sailing up and down the Thames, was no longer the best means of looking after them. Therefore, in 1845, it was resolved to build a church on a convenient site on shore. Ten years before this time the Sailors' Home had been built in Dock Street, and ground was appropriately found for the projected church—St. Paul's, Dock Street—close at hand.

With this church are connected names that are inextricably interwoven with English history. At the meeting held at the London Tavern on April 30th, 1845, when it was formally resolved to build the seamen's church, there were present Sir Edward Parry, Sir John Franklin, and other officers of the *Erebus* and *Terror*, then on the point of setting out on the most ill-fated of all Arctic expeditions. Sir Leopold McLintock has often worshipped there, and H.R.H. the Duke of Edinburgh has also shown his interest in the sailors' church. The church, which has been improved and beautified since its first erection, contains a beautiful window in memory of Sir John Franklin, and another in memory of Sir Edward Parry, representing his ship, the *Hecla*, when it was forced up against an iceberg in 1825. Another window is a memorial of three lads of sixteen, formerly pupils in the schools connected with the church, who were killed at sea. It represents the wreck of the *Gossamer*, off Prawle Point, near Dartmouth, on December 1st, 1868, when the captain, his newly-married wife, and nine sailors were drowned. "I married Captain Thompson and his wife," says Mr. Greatorex, the vicar of St. Paul's, "I gave them the communion the Sunday before they started, and in a few days came the news that they were drowned."

Mr. Greatorex could tell of many more incidents hardly less pathetic. He has been vicar of St. Paul's for twenty-six years now, and to his unwearying thought and energy is due the large extension of the work of the seamen's church. Dock Street and its surroundings did not bear the best of names at one time. Rosemary Lane and Ratcliff Highway were not inhabited by model characters, and one of Mr. Greatorex's early parishioners confessed, or boasted, that he had never held in his palm a single penny that he had earned honestly. For such a character as this any decided reformation was, in all probability, past hoping for; but where there are children there are always possibilities of good. So Mr. Greatorex has devoted himself greatly to bringing up the children of his flock in wholesome paths.

When the late Prince Consort laid the foundation-stone of the church he expressed the hope that now there was a church for the seamen and their wives, their children would not be forgotten. They have not been forgotten. Just at hand, in Wellclose Square, there have been erected schools, well-built and commodious, with an extent of garden that might be envied, which were opened by the Prince and Princess of Wales in 1870. In 1874 a further development took place in the erection of an infant nursery, which was opened by the Duke and Duchess of Edinburgh. By this means Mr. Greatorex gets hold of his children in their babyhood, when their mothers send them to the nursery to be kept while they are out working; they learn to walk in a railed-in cushioned space, generally known as "the fold;" they get their noon-day sleep in hammock-like swinging cots of stout linen; they dine there at a table about two feet high; are instructed in the art of using the spoon for the conveyance of food to their lips instead of the baby fingers they instinctively prefer. The walls of the nursery are hung with only good and pretty prints and illuminated texts. Nothing coarse or gaudy is permitted, for Mr. Greatorex has the idea—which it would be well if many well-meaning religious people shared—that to train the eye to appreciate beautiful things is a part of moral education. Children who have early been accustomed to good prints are not attracted by the woodcuts, equally atrocious in idea and execution, that adorn (?) the *Police News*.

From the nursery the children, as they grow older, go to the schools. The instruction there is of the ordinary Board-school type, but thorough, because the classes are not over-large; and a very important addition to what the children gain is the substantial dinner that forms part of the daily routine. Not the "self-supporting penny dinner," which Mr. Greatorex holds in something like scorn. A child's dinner is not, or should not be, merely a meal to keep it from feeling hungry at the moment. It should furnish material for the sound and ample growth of healthy limbs and organs. It is a very exceptional penny dinner that will do this; not, as a rule, one in which the price is equivalent to the cost. Mr. Greatorex finds he must spend more than a penny on these meals if they are to result in the firm flesh and rosy cheeks that give the lie to all that has been written of the dreary East End.

He does not, however, consider the east either dreary or unhealthy. Wellclose Square, indeed, has an interest of its own. It was the first square ever built in London; the Spanish, Danish, and Scandinavian Ambassadors used to live there, and their handsome houses are still standing, though marred by being cut up into small tenements. It has also records going back beyond the days of ambassadors to England. When the foundations of the schools were being dug in the square garden, the workmen came on a Roman sarcophagus containing the remains of two children. The lid was broken in, the coffin wholly filled with earth, but the sarcophagus itself was sound, and is preserved in the grounds of the school.

Mr. Greatorex is a man of resource; and it struck him that in the infant nursery there was a good deal of wall and ceiling space that might be put to some use. He improved it by a dado of polished wood and a stove, and transformed it into a cottage hospital, where children, tended by those whom they already know and trust—Mr. Greatorex himself has studied medicine, and acts to some extent as house surgeon to the hospital—recover far sooner than they would in their own homes, sooner even than they would in a general hospital. Besides the organisations already described, there is a Clothed Scholars Fund, for the fatherless children of seamen, and among seamen's families there will always be many fatherless; mother's meetings, a penny bank, a lending library, and the usual parochial visiting agencies. All these do infinite good; but they cannot be carried on without money, and in a parish where not more than thirty inhabitants have incomes reaching two pounds a-week, and the large majority do not come near that sum, money must be sought for outside. If the work were more widely known, surely the money would be forthcoming; surely the unwearying tender-hearted man who has laboured with such patience, energy, and success among our sailors and their families for twenty-six years, would not be kept so burdened with pecuniary anxieties, would not be harassed with the fear that he may have to give up some of his beneficent agencies. Let those who are interested in poor Jack, and feel some kindness towards him and those he loves, visit the Rev. Dan Greatorex at Dock Street, and then judge for themselves what their duty is.

